# Efficacy of oral afoxolaner for the treatment of canine generalised demodicosis

**DOI:** 10.1051/parasite/2016014

**Published:** 2016-03-24

**Authors:** Frédéric Beugnet, Lénaïg Halos, Diane Larsen, Christa de Vos

**Affiliations:** 1 Merial S.A.S. 29 avenue Tony Garnier 69007 Lyon France; 2 Clinvet International (Pty) Ltd PO Box 11186 9321 Universitas South Africa

**Keywords:** *Demodex canis*, Demodicosis, Treatment, Afoxolaner, NexGard^®^, Dog

## Abstract

The efficacy of oral treatment with a chewable tablet containing afoxolaner 2.27% w/w (NexGard^®^, Merial) administered orally was assessed in eight dogs diagnosed with generalised demodicosis and compared with efficacy in eight dogs under treatment with a topical combination of imidacloprid/moxidectin (Advocate^®^, Bayer). Afoxolaner was administered at the recommended dose (at least 2.5 mg/kg) on Days 0, 14, 28 and 56. The topical combination of imidacloprid/moxidectin was given at the same intervals at the recommended concentration. Clinical examinations and deep skin scrapings were performed every month in order to evaluate the effect on mite numbers and the resolution of clinical signs. The percentage reductions of mite counts were 99.2%, 99.9% and 100% on Days 28, 56 and 84, respectively, in the afoxolaner-treated group, compared to 89.8%, 85.2% and 86.6% on Days 28, 56 and 84 in the imidacloprid/moxidectin-treated group. Skin condition of the dogs also improved significantly from Day 28 to Day 84 in the afoxolaner-treated group. Mite reductions were significantly higher on Days 28, 56 and 84 in the afoxolaner-treated group compared to the imidacloprid/moxidectin-treated group. The results of this study demonstrated that afoxolaner, given orally, was effective in treating dogs with generalised demodicosis within a two-month period.

## Abstract (fr)

L’efficacité d’un traitement par comprimés appétents contenant de l’afoxolaner 2.27 % w/w (NexGard^®^, Merial), administré oralement, a été évaluée chez 8 chiens atteints de démodécie généralisée, et comparée avec l’efficacité chez 8 chiens d’une application topique de la combinaison imidaclopride/moxidectine (Advocate^®^, Bayer). L’afoxolaner était administré à la dose recommandée (au minimum 2.5 mg/kg) aux jours 0, 14, 28 et 56. La combinaison imidaclopride/moxidectine était administrée aux mêmes intervalles à la dose recommandée. Les examens cliniques et les raclages cutanés profonds ont été réalisés chaque mois pour évaluer la réduction du nombre d’acariens et la résolution des signes cliniques. Les pourcentages de réduction du nombre d’acariens ont été de 99.2 %, 99.9 % et 100 % aux jours 28, 56, et 84, respectivement, dans le groupe traité avec l’afoxolaner, comparé à 89.8 %, 85.2 %, et 86.6 % aux jours 28, 56, et 84 pour le groupe traité avec imidaclopride/moxidectine. Les signes dermatologiques se sont considérablement améliorés du jour 28 au jour 84 dans le groupe traité avec l’afoxolaner. La réduction du nombre d’acariens a été significativement plus importante aux jours 28, 56, et 84 dans le groupe traité avec l’afoxolaner par rapport au groupe traité avec imidaclopride/moxidectine. Les résultats de cette étude démontrent que l’afoxolaner, administré oralement, est efficace dans le traitement de la démodécie généralisée chez le chien dans une période de deux mois.

## Introduction

Demodicosis is a parasitic skin disease of dogs caused by proliferation of mites of the species *Demodex canis* Leydig, 1859 [17, 29]. A small number of mites may be considered a normal component of the dog’s skin fauna, but their proliferation may lead to a serious disease. Puppies acquire mites in the first days of their life through direct skin contact from their mother [19, 20]. Three morphologically different types of *Demodex* have been described in dogs. They have been called *Demodex canis* (medium length opisthosoma), *Demodex injai* (long opisthosoma) and *Demodex cornei* (short opisthosoma) [27]. There is controversy as to whether these types may be related to development stages, host response and natural variability, or to separate species [25]. By comparing the mitochondrial 16S rDNA, Sastre et al., 2012, concluded that *Demodex canis* and *D. injai* were two different species, with a genetic distance of 23.3% obtained in their study. But the short-bodied *Demodex* mite so-called *D. cornei* appeared to be a morphological variant of *D. canis* [27]. Later in 2012, Rojas et al., by using both the cytochrome oxidase I gene and 16S rDNA, concluded differently. From genetic distance and divergence data they considered *D. canis*, *D. injai* and *D. cornei* to be polymorphisms of the same species [25]. A final consensus on taxonomy will require molecular testing and until then, *Demodex canis* is the only valid species [24]. Published data indicate similar efficacy of reported treatments regardless of the *Demodex* morphotype [19, 20].

Canine demodicosis is classically divided into two clinical manifestations by dermatologists: localised and generalised demodicosis. The localised form appears as patches of alopecia and mild erythema in young dogs. It can regress spontaneously without treatment. The generalised form of demodicosis is more severe and can even be fatal in case of bacterial secondary infection. It may develop from the localised condition or occur spontaneously in young and subadult dogs, but also in older animals especially those under severe stress or with underlying diseases [12]. The definition of localised versus generalised demodicosis has been a matter of debate [19, 20]. A recent Committee of Experts considered demodicosis as localised if there are no more than four lesions with a diameter of up to 2.5 cm [12, 19, 20]. Nevertheless, this needs to be adapted to breed and each clinical situation by the practitioner, i.e. three lesions of 2 cm in diameter in a young Chihuahua may be considered generalised demodicosis, while five lesions of 3 cm could be diagnosed as localised demodicosis in an English Mastiff. Canine generalised demodicosis is frequently seen in practice and is characterised by five or more affected areas or by lesions covering an entire region of the body, and/or pododemodicosis involving two or more paws. The affected areas are erythematous, with comedones, hair loss, follicular papules to pustules and scales. Lymphadenopathy is commonly associated with the disease and secondary bacterial infections are frequent. Although some young dogs with an early generalised form can self-cure, it is impossible to clinically ascertain which animals will progress to the more severe state [23]. Generalised demodicosis in young adult dogs is considered to be related to immune deficiency with a genetic base. To decrease the prevalence in pure breed dogs, it has been recommended to avoid breeding dogs with generalised demodicosis. In recent international treatment guidelines, it is recommended that each dog requiring acaricidal therapy should be neutered [12, 20].

The diagnosis is typically based on clinical signs and is confirmed by the presence of mites in deep skin scrapings. Although *Demodex* mites are part of the normal microfauna, it is uncommon to find the mites, even by performing several deep skin scrapings. If a mite is found, this should raise suspicion and additional skin scrapings should be performed. Finding more than one mite is strongly suggestive of clinical demodicosis [12, 19, 20].

Generalised demodicosis is a very challenging disease to treat effectively. Only a few drugs and formulations are registered, with variable efficacy [20].

Amitraz (Ectodex^®^, Mitaban^®^), applied topically as a rinse or sponge on, has been approved for the treatment of canine generalised demodicosis in many countries for decades [7]. Several amitraz-based protocols have been described in which amitraz is applied at various concentrations and frequencies. Efficacy data are variable, treatment protocols are time-consuming and there may be safety issues. Recently, topical formulations containing amitraz combined either with the insecticide metaflumizone (ProMeris duo^®^), or with the insecticide-acaricide fipronil and the insect growth regulator S-methroprene (Certifect^®^), have shown efficacy [8, 11]. Their easier administration helps improve owner compliance and therefore increases the rate of success. Nevertheless, safety issues have limited the use of both ProMeris Duo^®^ and Certifect^®^, especially the risk of triggering pemphigus foliaceus reactions [3, 21].

Protocols based on daily to weekly oral or subcutaneous injections of macrocyclic lactones including ivermectin, doramectin and moxidectin at high doses are reported to provide variable efficacy, but may also have potential for toxicity, especially in dogs carrying MDR-1 gene mutations (P-glycoprotein deficiency), especially including Collie breeds [18, 20, 22]. Daily oral milbemycin oxime at a minimum dose of 0.5 mg/kg (Interceptor^®^) is registered in some countries for the treatment of canine demodicosis [15]. It provides good results but its cost, especially for treating large-breed dogs, appears to be a limiting factor for its use. Moxidectin, combined with the insecticide imidacloprid (Advocate^®^, Bayer), in a topical formulation that has shown efficacy in several studies [10, 14, 22] and has been approved in several European countries has therefore been used as a positive control in studies assessing the efficacy of new drugs, following registration agency guidelines. However, efficacy rates seem to be more variable under field conditions, and it appears to be more efficacious in juvenile dogs with milder forms of the disease [20]. Bi-weekly or weekly treatments showed better results than monthly administration [19, 20].

Whatever the choice of the antiparasitic drug by the veterinarian, the duration of treatment for demodicosis is usually 3 months or more.

Recently, a new class of insecticides/acaricides, the isoxazolines, have demonstrated very good efficacy against fleas and ticks [28]. The efficacy of fluralaner (Bravecto^®^), a molecule belonging to this group, against canine demodicosis has recently been demonstrated [9]. Fluralaner was given once orally at the minimum dose of 25 mg/kg and the dogs were evaluated for 3 months for efficacy based on mite numbers and clinical lesion scores. After a single oral administration of fluralaner chewable tablets, mite numbers in skin scrapings were reduced by 99.8% on Day 28 and by 100% on Days 56 and 84. Statistically significantly (*P* ≤ 0.05) fewer mites were found on Days 56 and 84 on the fluralaner-treated dogs compared to imidacloprid/moxidectin-treated dogs.

Afoxolaner is another isoxazoline administered monthly to protect dogs against fleas and ticks (NexGard^®^) [6, 13, 28]. It is administered at a minimum dose of 2.5 mg/kg, and comparative studies have shown that three monthly administrations provided at least similar results compared to fluralaner administered at considerably higher dose (25 mg/kg) once against fleas and ticks [1, 2]. Based on these observations, and following feedback from veterinarians having tried the treatment, the purpose of the present study was to assess the efficacy of oral administration of afoxolaner (NexGard^®^), against generalised canine demodicosis.

## Materials and methods

### Study design and treatment

The design and conditions of this study were approved by the local Animal Welfare Ethics Committee in accordance with Good Clinical Practice by the European Agency for the Evaluation of Veterinary Medicinal Products (CVMP/VICH GL9, July 2000; CVMP/VICH GL19, July 2001) [5]. All dogs belonged to private owners who signed an owner consent for the inclusion of their dogs in the study. The study followed a randomised block design. The 16 dogs included were ranked within sex in descending order of individual pre-administration (Day −2) mite counts and subsequently blocked into eight blocks of two dogs each. Dogs were then allocated to treatment groups, and all evaluations of efficacy were performed by personnel in blinded conditions. The study was conducted on two groups of eight dogs each: group 1 dogs were treated with the topical combination of imidacloprid/moxidectin and group 2 dogs were treated orally with afoxolaner (Table 1). All dogs were weighed and treated either topically with Advocate^®^ (imidacloprid 10% w/v and moxidectin 2.5% w/v spot on solution) or orally with NexGard^®^ (2.27% w/w chewable tablets) in accordance with European label instructions (Table 2).

**Table 1.** Summary of the study schedule.

Acclimatisation	Ranking into groups	Administration of NexGard^®^ or Advocate^®^	
Days −14 to −1	Day −2/−1	Days 0, 14, 28 and 56	
Mite counts and clinical symptom assessments (including Photographic documentation, Supplementary Material)	Skin Biopsies	Clinical examinations	Body weight determination
Days −2 or −1, 28, 56 and 83 or 84	Days −7 and 34	Days −14, −7, −2 or −1, 14, 28, 56 and 83 or 84	Days −14, −7, −2 or −1, 13, 27, 55 and 83 or 84

**Table 2.** Dosage recommendation for NexGard^®^ and Advocate^®^ according to European labels.

Group	Sample size	Product	Active ingredient(s)	Dose rate
1	8 dogs	Advocate^®^	Imidacloprid 10% w/v and moxidectin 2.5% w/v spot on solution	> 1 kg to 4 kg: one 0.4 mL tube/dog; > 4 kg to 10 kg: one 1.0 mL tube/dog; > 10 kg to 25 kg: one 2.5 mL tube/dog; > 25 kg up to 40 kg: one 4.0 mL tube/dog
2	8 dogs	NexGard^®^	Afoxolaner (2.27% w/w)	2 kg to 4 kg: 11 mg afoxolaner; > 4 kg to 10 kg: 28 mg afoxolaner; > 10 kg to 25 kg: 68 mg afoxolaner; > 25 kg to 50 kg: 136 mg afoxolaner.

The European approved label instructions for the use of Advocate^®^ recommend monthly use with the possibility of increasing the duration and/or the frequency of application: “The administration of a single dose every 4 weeks for 2–4 months is efficacious against *Demodex canis* and leads to a marked improvement of clinical signs particularly in mild to moderate cases. Especially severe cases may require more prolonged and more frequent treatment. To achieve the best possible response in these severe cases, at the discretion of the veterinarian, Advocate can be applied once a week and for a prolonged time. In all cases it is essential that the treatment should be continued until skin scrapings are negative on at least two consecutive monthly occasions.” http://www.ema.europa.eu/ema/index.jsp?curl=pages/medicines/veterinary/medicines/000076/vet_med_000102.jsp&mid=WC0b01ac058001fa1c.

The dogs were treated on Days 0, 14, 28 and 56. Mite counts and clinical assessments were performed on Days 2, 28, 56 and 84. All dogs were aged > 6 months, male or female and were healthy at the initiation of the study, except for clinical signs of generalised demodicosis as determined by a veterinarian on Days −14, −7 and −2/−1.

Dogs were included in the study if they presented clinical signs of *Demodex canis* infestations which may include erythema, hair loss, comedones, follicular casts, scales and crusts. Deep skin scrapings were performed on Day −1 to confirm the presence of *Demodex* spp. mites. All the dogs presented signs of generalised demodicosis (i.e. more than five spots, pododemodicosis involving two or more paws, or an entire body region). The females were not pregnant. Finally, the dogs had not been treated with glucocorticoid therapy or any ectoparasiticide or macrocyclic lactone for at least 12 weeks prior to Day 0, as far as it could be reasonably established by verbal communication with the owners.

The included dogs weighed 4.5–15.1 kg, seven males and nine females.

The dogs were acclimatised for at least 14 days before treatment, and any medication given to the animals during the acclimatisation period was recorded. The dogs were leased from various owners during the month preceding the study and moved back to their household after the study. They originated from Bloemfontein (South Africa) and its suburbs, and were privately owned, not stray dogs. They were enrolled after diagnosis of generalised demodicosis. Based on the experience of the authors and past studies, to avoid cases of improvement of the condition after enrolment, especially due to the change in food during the experimental conditions, their clinical status was followed during the 2 weeks before start of the study in order to remove the dogs that would have improved naturally.

The dogs were contained in cages consisting of a 1.69 m × 0.7 m enclosed sleeping area, with under floor heating, and an outside run of 1.69 m × 3.0 m. A roof covered the kennels and the dogs were therefore not exposed to rain. The animals were exposed to ambient temperatures and lighting was provided by natural sunlight. They were fed once a day and water was provided in stainless steel bowls and replenished at least twice daily.

Trained personnel under the supervision of a veterinarian were responsible for the health of the animals. Abnormal signs were reported from daily observations, post-administration observations and scheduled clinical examinations. Authorised concurrent medications included antimicrobials, and vitamin and mineral supplements. The use of concomitant veterinary care and therapy was recorded.

Dogs were fed with standard commercially available animal food at the recommended rates.

### Assessment of efficacy

A veterinarian, or qualified member of personnel under the supervision of a veterinarian, conducted a clinical examination on all dogs on Days −7, −2, 14, 28, 42, 56, 70 and 84. All the animals were observed daily from Day −7 to Day 84 for general health and from Days 0 to 84 for clinical signs of adverse events to treatment administration.

Efficacy evaluation was based on the decrease in *Demodex* spp. mites relative to baseline and the resolution of clinical signs.

#### Mite counts (primary criterion)

Deep skin scrapings from five sites were taken on the days of clinical examination. Skin scraping sites were recorded and these sites and/or sites of new lesions were scraped at each subsequent examination. Skin scrapings were made with a blade so that capillary oozing occurred.

The scraping was transferred to a marked (animal ID, group and body region) microscope slide containing mineral oil and was examined under a stereomicroscope for the presence of live or dead *Demodex* spp. mites. The number of mites counted in each scraping was recorded separately.

### Clinical symptom evaluation

The clinical symptoms and the extent of demodectic lesions on each dog were assessed on the same days during which scrapings were made and recorded on a standardised form. The following parameters were assessed for each dog and sketched on a silhouette (left- and right-hand side) of a dog:

- body areas covered by casts, scales and crusts;
- body areas with hair loss (1 = slight thinning of hair; 2 = conspicuous hair loss; 3 = no hair);
- body areas with erythema;
- estimated hair re-growth (Table 3).
  **Table 3.** Semi-quantitative assessment used to assess hair growth.

Score	Description
1	Body areas with hair re-growth 0–50% compared to that recorded during the pre-administration assessment
2	Body areas with estimated hair re-growth > 50% ≤ 90% compared to that recorded during the pre-administration assessment
3	Body areas with estimated hair re-growth > 90% compared to that recorded during the pre-administration assessment

Coloured photographs to illustrate the extent of lesions and the resolution of lesions were taken for each dog before treatment administration (Day −2) and on Days 28, 56 and 84.

### Statistical analysis

The primary assessment variable in this study was the decrease in mite counts (immature and adult live mites combined).

The individual percentage decrease from the pre-administration mite count to the post-administration mite count in each dog on each assessment day was calculated by:

Decrease% (individual)=(Pre-administration-Post-administration)/Pre-administration×100

where

Pre-administration = the mite counts of a dog prior to the first treatment, and

Post-administration = the mite counts of a dog after the relevant treatment.

The average percent reduction in mite counts for the group was calculated by:

Decrease% (group)=([GMPre-administration-GMPost-administration]/GMPre-administration)×100

where

GMPre-administration = the geometric mean (GM) of the pre-administration mite counts, and

GMPost-administration = the geometric mean of the post-administration mite counts.

The number of mites on each assessment day and each group were tabulated with the following descriptive statistics: mean, median, standard deviation (SD), geometric mean, minimum and maximum.

### Clinical signs and symptoms

Data recorded during clinical assessments on casts, scales, crusts and area(s) of hair loss and erythema were summarised in tables for each dog. Overall changes in clinical appearance were also reported in these tables by pre- and post-administration photographs taken from each dog. These tables showed the overall extent and resolution of lesions for each dog.

As a secondary criterion, the number of dogs affected by erythema, casts, scales and crusts was compared between the pre-administration and different post-administration assessment days. A semi-quantitative assessment of hair re-growth was also conducted and a score awarded to each dog on the different post-administration assessment days (Table 3). Photographic documentation was used to highlight changes in skin condition.

The pre-administration and post-administration mite counts were compared by ANOVA with administration (pre or post) and animal effects on mite count data. SAS Version 9.3 TS Level 1M2 was used for all the statistical analyses. The level of significance of the formal tests was set at 5%, all tests were two-sided.

## Results

### Antiparasitic efficacy

All the dogs were positive for the presence of mites prior to the first treatment. For both treated groups on all post-treatment assessment days, the mite counts were statistically significantly reduced (*p* < 0.05) compared to the counts at initiation (Table 4). The geometric mean number of mites recorded from the dogs prior to treatment administration was 808.1 in group 1 (Advocate^®^) and 650.8 in group 2 (Nexgard^®^), indicating a vigorous pre-existing *Demodex* spp. infestation in each group. No significant differences (*p* = 0.8103) were recorded between the geometric mean number of mites between groups prior to treatment administration.

**Table 4.** Mite count reduction in treated groups (based on geometric means).

	Group 1 – Imidacloprid/moxidectin			Group 2 – Afoxolaner		
Day	Geo mean	Reduction (%)	*p*-value	Geo mean	Reduction (%)	*p*-value
0	808.1	/	/	650.8	/	/
28	82.4*	89.8	0.0008	5.3*	99.2*	<.0001
56	119.9*	85.2	0.0038	0.6*	99.9*	<.0001
84	108.5*	86.6	0.0025	0*	100*	<.0001

* Group 2 differed statistically significantly (*p* < 0.05) from group 1.

After one month, the geometric mean number of *Demodex* spp. mites recorded from the dogs in group 1 (imidacloprid/moxidectin) ranged from 82.4 to 119.9, significantly fewer (*p* < 0.05) than during the pre-treatment evaluations. The geometric mean number of *Demodex* spp. mites recorded from the dogs in group 2 (afoxolaner) ranged from 0.0 to 5.3 after one month, significantly fewer (*p* < 0.05) than during the pre-treatment evaluations. On Day 84, no live mites were recorded for any dog in the Nexgard^®^-treated group. Significantly fewer mites (*p* < 0.05) were recorded from the dogs in the afoxolaner-treated group compared to the imidacloprid/moxidectin-treated group at all post-treatment assessment days. The efficacy of Nexgard^®^ ranged from 99.2% to 100% during the assessment period.

### Clinical efficacy

Erythematous papules were recorded from only one dog in each treatment group. These had completely resolved following treatment in the Nexgard^®^-treated group by Day 28 and in the Advocate^®^-treated group by Day 84. The occurrence of crusts, casts and scales was reduced in both treatment groups, but had not resolved in all animals. By Day 84, 50% of the Advocate^®^-treated animals still had crusts or scales, compared to 25% of the animals in the Nexgard^®^-treated group (Table 5, Photographic documentation, Supplementary Material).

**Table 5.** Resolution of clinical signs and symptoms during the assessment period of 90 days.

Day	Clinical sign	Group 1 (Imidacloprid/moxidectin)	Group 2 (afoxolaner)
		Number of dogs (%)	Number of dogs (%)
−2/−1	Casts	1/8 (12.5)	1/8 (12.5)
	Crusts	6/8 (75)	7/8 (87.5)
	Scales	4/8 (50)	1/8 (12.5)
	Erythematous papules	1/8 (12.5)	1/8 (12.5)
28	Casts	1/8 (12.5)	1/8 (12.5)
	Crusts	7/8 (87.5)	4/8 (50)
	Scales	5/8 (62.5)	1/8 (12.5)
	Erythematous papules	0/8 (0)	0/8 (0)
56	Casts	0/8 (0)	0/8 (0)
	Crusts	5/8 (62.5)	2/8 (25)
	Scales	4/8 (50)	2/8 (25)
	Erythematous papules	1/8 (12.5)	0/8 (0)
84	Casts	0/8 (0)	0/8 (0)
	Crusts	3/8 (37.5)	2/8 (25)
	Scales	4/8 (50)	2/8 (25)
	Erythematous papules	0/8 (0)	0/8 (0)

A marked improvement in hair re-growth was observed in all the dogs in both treated groups from eight weeks after initial treatment onwards (Table 6).

**Table 6.** Percentage hair re-growth during the assessment period.

Day	Estimated percentage of hair re-growth					
	Group 1 (Imidacloprid/moxidectin) (number of dogs/eight dogs per group)			Group 2 (Afoxolaner) (number of dogs/eight dogs per group)		
	0–50%	50–90%	>90%	0–50%	50–90%	>90%
28	6/8	2/8	0/8	6/8	1/8	1/8
56	3/8	3/8	2/8	2/8	3/8	3/8
84	1/8	4/8	3/8	1/8	4/8	3/8

### Health observations

No adverse events related to treatments were observed during the study.

## Discussion and conclusion

The present study demonstrates that treatments with an oral formulation of afoxolaner (NexGard^®^) resulted in a rapid reduction in mite numbers and a marked improvement in clinical signs in all dogs. A significant portion of afoxolaner-treated dogs (7/8) had no mites in their skin scrapings at Day 84. In contrast, seven of the eight dogs in the imidacloprid/moxidectin group were still infested on Day 84.

The spot on combination imidacloprid/moxidectin was used at a two-week interval and then monthly. The first publication on its efficacy by Fourie in 2007 demonstrated efficacy with monthly applications [11]. Nevertheless, the success of treatments seems to be more variable under field conditions in Europe and therefore the European labelling was modified and stated that both duration and frequency should be adapted, up to weekly administration.

The main reason to explain a greater efficacy under the experimental studies was related to the recruitment of the owned dogs which could be in poor health conditions at inclusion and then received good care, including good quality food, deworming and housing [10, 11]. Having higher quality care can increase the immune status and therefore the response to treatment against *Demodex*. To limit this effect, the most recent efficacy studies did recruit owned dogs having better care at the origin (especially regarding diet) and most importantly acclimatised the dogs in advance (2 weeks or more) before the start of the study to be able to exclude dogs with significant improvement of their condition before allocation. The baseline mite count was also determined at the time of treatment, not at the time of recruitment. The acclimatisation of dogs in advance was also performed for the studies assessing Certifect^®^ and Bravecto^®^ [8, 9].

The average mite count in the imidacloprid/moxidectin group at Day 56, still 108.5 mites, may be considered further evidence for the accuracy of the data presented in this study. A weekly application would probably have given better results.

The quick clinical efficacy of monthly administrations of NexGard^®^ under field conditions in both Europe and the USA has been reported by several dermatology speakers (Rosenkrantz W., oral communication at NAVC 2016, Orlando [26]; Ferrer L., V Feline Medicine Congress GEMFE-AVEPA, 2016, Alicante (Spain)), which may indicate the accuracy of the actual experimental data. The sensitivity of skin scrapings to detect remission of demodicosis has sometimes been challenged [19, 20]. As the life cycle of the mite extends over a period of 18–24 days, and considering that scrapings are performed on a limited area of the lesions, a single negative skin scraping should generally not be considered as an indication of complete remission. Remission should rather be determined based on two consecutive negative skin scrapings done at a one-month interval [8]. In the present study, seven out of eight NexGard^®^-treated dogs had two successive negative skin scrapings at a one-month interval, indicating that treatments at appropriate intervals can provide remission of the disease [9, 20]. The significant clinical improvement seen on all dogs is another sign of effective treatment. It is known that even without mites, the lesions will disappear slowly in some dogs due to the time needed for skin to fully recover.

The Committee for Medical Products for Veterinary Use (CVMP) guideline “Guideline for the testing and evaluation of the efficacy of anti-parasitic substances for the treatment and prevention of tick and flea infestations in cats and dogs” states that at least six animals should be used per group [5]. Eight dogs per group were used in this study to increase the validity of the results.

The bi-weekly treatments with NexGard^®^ at Days 0, 14 and then 28 were based on the pharmacokinetic properties of afoxolaner, in order to rapidly achieve a steady-state concentration in the blood [16]. Treating at the label dose three times at two-week intervals was considered to be safe because this schedule was performed in the NexGard^®^ target animal safety study conducted for its registration in the USA and Europe [4, 6]. In that study, no adverse events related to the treatment were observed during the 84-day period.

Following the results in this controlled study, the efficacy of monthly administrations of NexGard^®^ should be further assessed under field conditions in veterinary clinics.

In conclusion, the high level of efficacy achieved with the afoxolaner-based chewable tablets offers new perspectives to veterinarians for the control of demodicosis. It provides a new solution that combines safety, efficacy and ease of use for improved owner compliance.

## Conflict of interest

This clinical study was funded by Merial S.A.S., 29 avenue Tony Garnier, 69007 Lyon of which Frédéric Beugnet and Lénaïg Halos are employees.

ClinVet, of which Christa de Vos is an employee, is an independent South African Contract Research Organisation contracted to conduct the study.

All authors voluntarily publish this article and have no personal interest in these studies other than publishing the scientific findings that they have been involved in via planning, initiating, monitoring and conducting the investigations and analysing the results.

## Disclaimer

NEXGARD^®^ is a registered trademark of Merial. All other brands are the property of their respective owners.

This document is provided for scientific purposes only. Any reference to a brand or a trademark herein is for informational purposes only and is not intended for a commercial purpose or to dilute the rights of the respective owner(s) of the brand(s) or trademark(s).

## Supplementary Material

Photographic documentation
