# Supplementary material for: Efficacy of oral afoxolaner for the treatment of canine generalised demodicosis
Source: Parasite. 2016 Mar 24;23:14. doi: 10.1051/parasite/2016014 (PMC4807374; doi:10.1051/parasite/2016014)
Supplement: Photographic documentation [file parasite-23-14-s1.pdf]

PHOTOGRAPHIC DOCUMENTATION

Animal ID	5D1 C97
Treatment	NexGard®

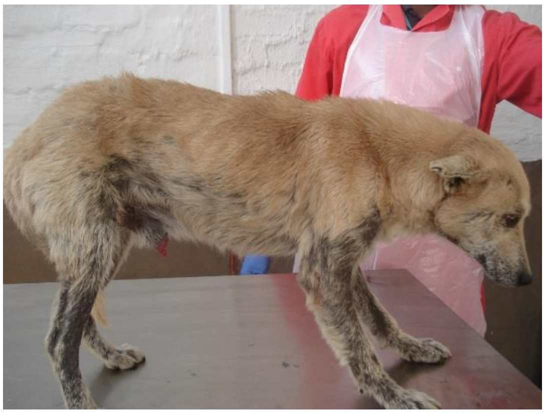

Day	Pre-treatment
Mite count	1741

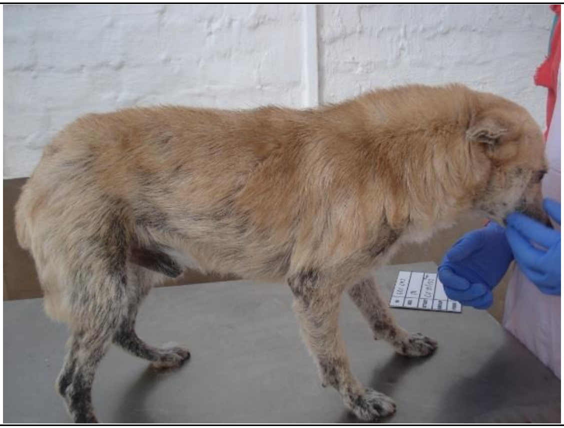

Day	28
Mite count	1

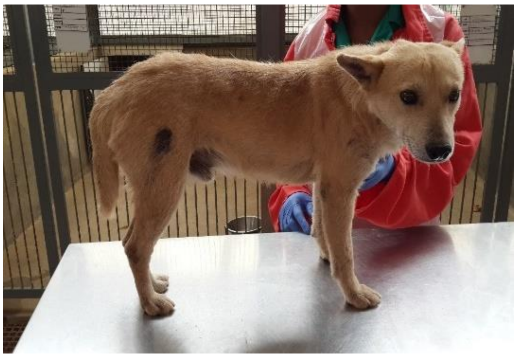

Day	56
Mite count	0

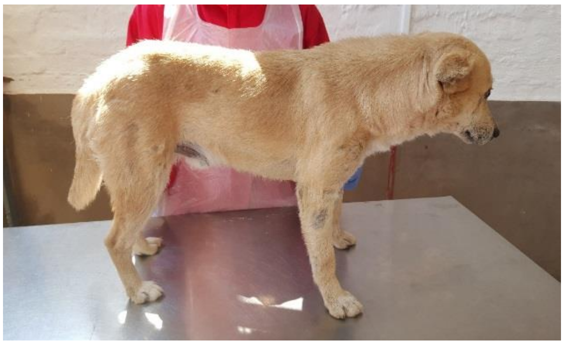

Day	84
Mite count	0

PHOTOGRAPHIC DOCUMENTATION

Animal ID	5C8 2AA
Treatment	NexGard®

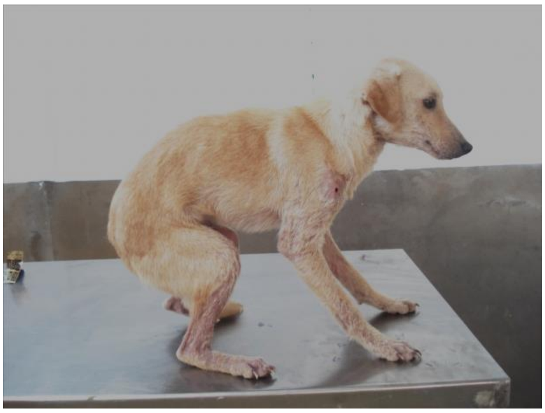

Day	Pre-treatment
Mite count	4637

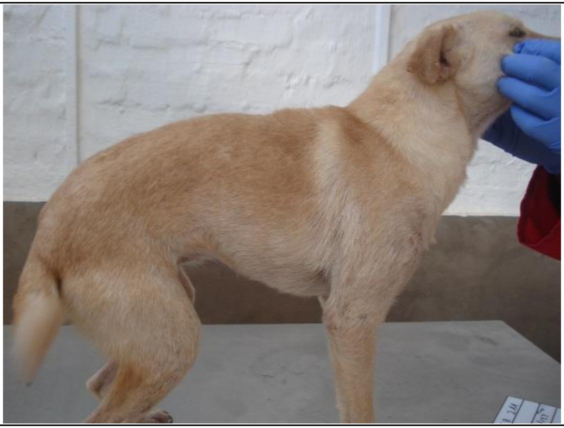

Day	28
Mite count	15

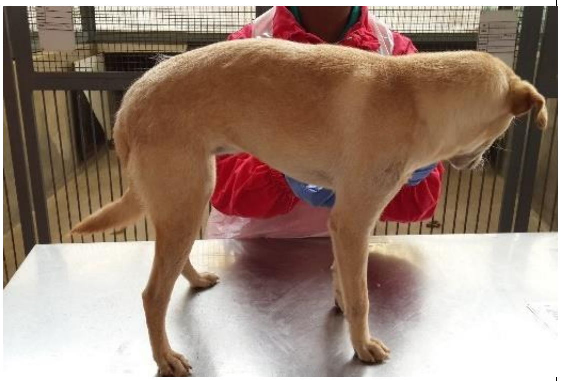

Day	56
Mite count	0

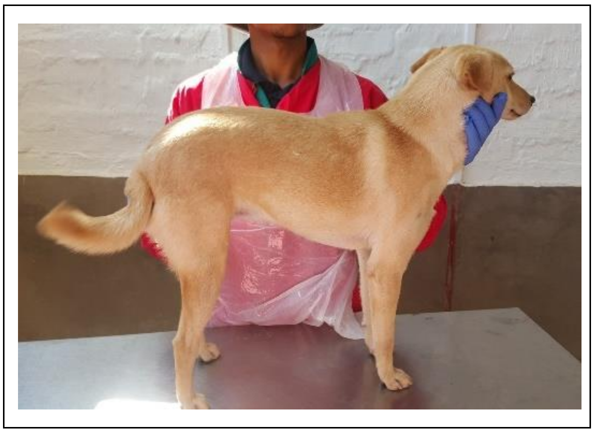

Day	84
Mite count	0

PHOTOGRAPHIC DOCUMENTATION

Animal ID	698 376
Treatment	NexGard®

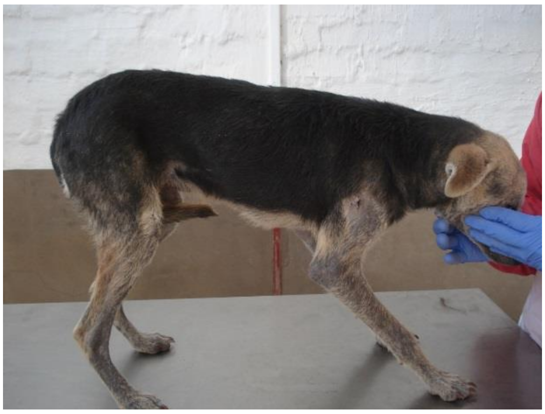

Day	Pre-treatment
Mite count	203

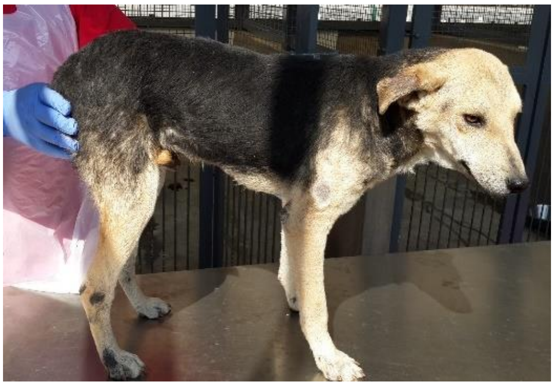

Day	28
Mite count	0

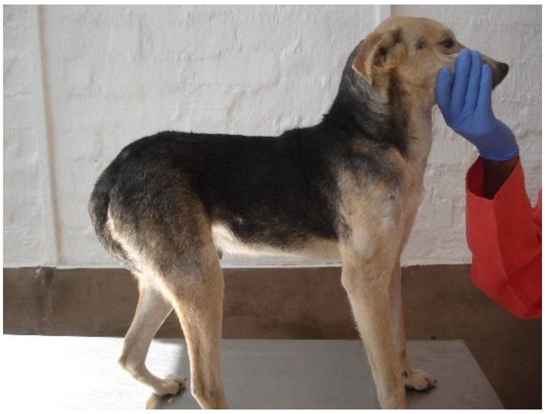

Day	56
Mite count	0

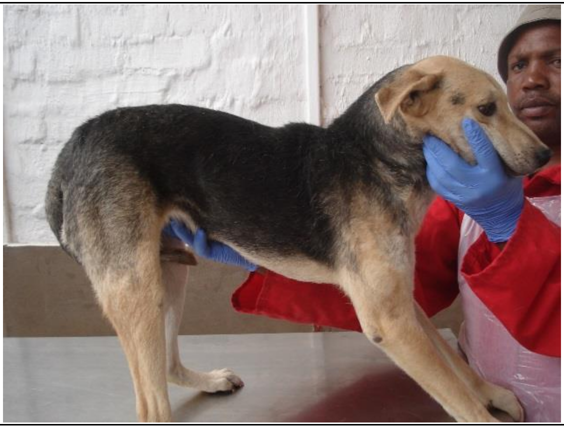

Day	84
Mite count	0

PHOTOGRAPHIC DOCUMENTATION

Animal ID	698 38D
Treatment	NexGard®

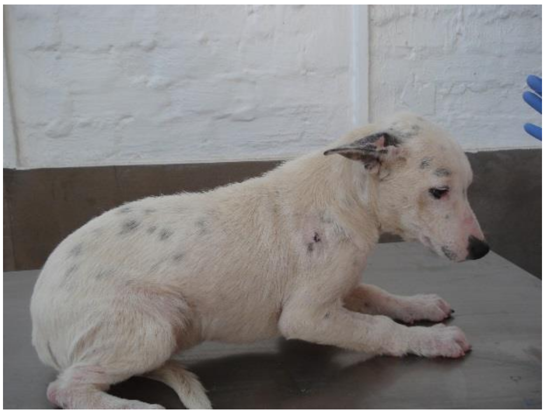

Day	Pre-treatment
Mite count	8

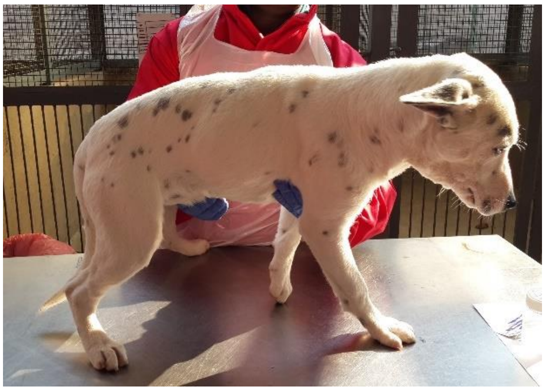

Day	28
Mite count	0

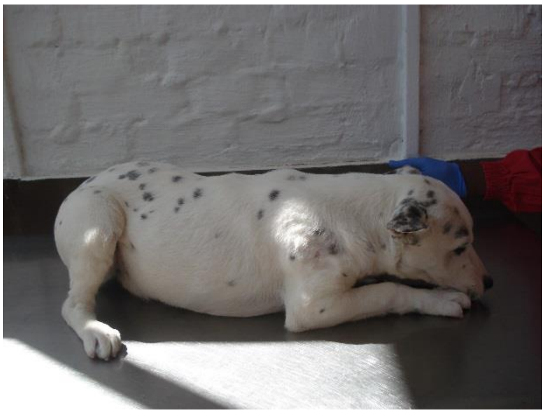

Day	56
Mite count	1

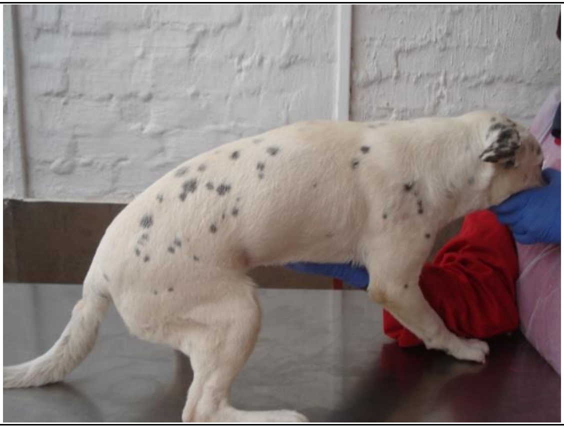

Day	84
Mite count	0

PHOTOGRAPHIC DOCUMENTATION

Animal ID	698 0FF
Treatment	NexGard®

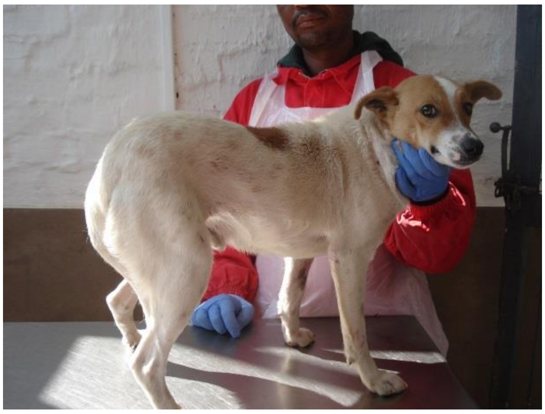

Day	Pre-treatment
Mite count	566

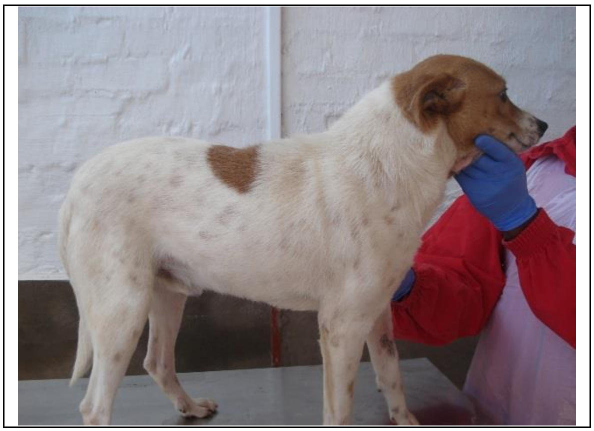

Day	28
Mite count	19

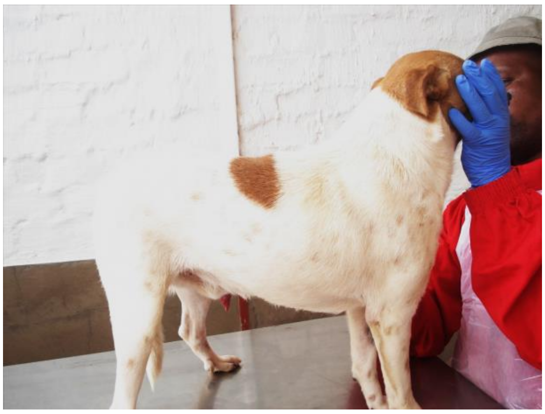

Day	56
Mite count	0

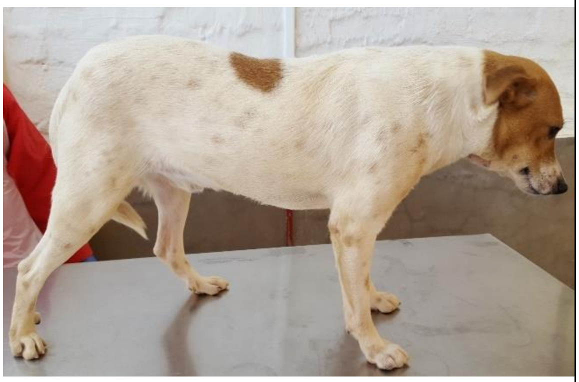

Day	84
Mite count	0

PHOTOGRAPHIC DOCUMENTATION

Animal ID	5A8 C5A
Treatment	NexGard®

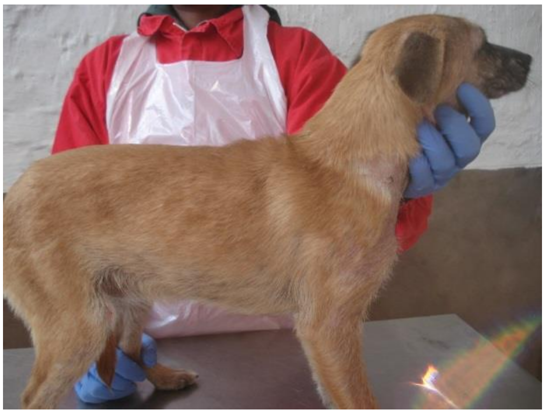

Day	Pre-treatment
Mite count	3121

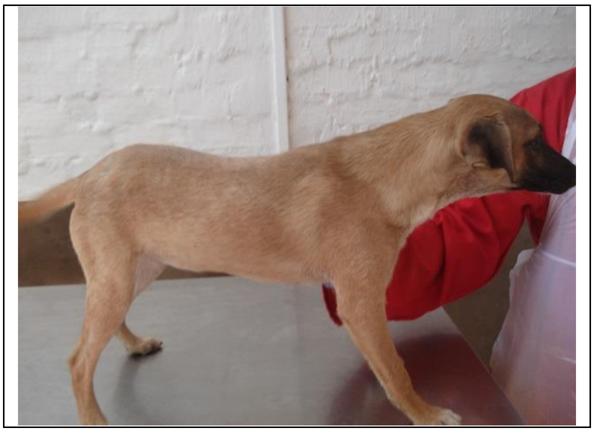

Day	28
Mite count	152

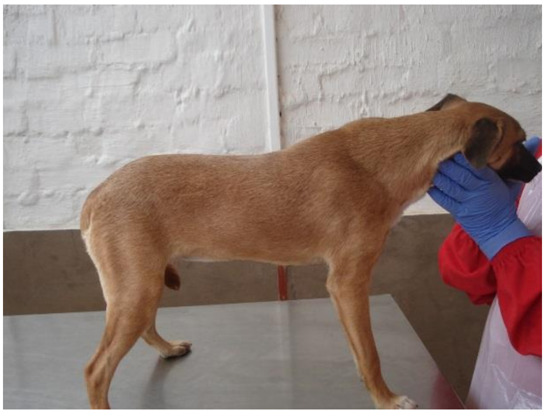

Day	56
Mite count	23

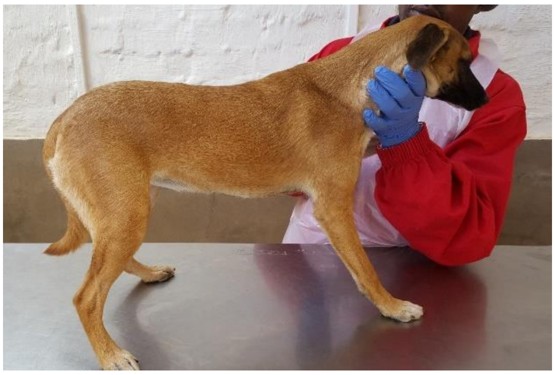

Day	84
Mite count	0

PHOTOGRAPHIC DOCUMENTATION

Animal ID	6D4 1A0
Treatment	NexGard®

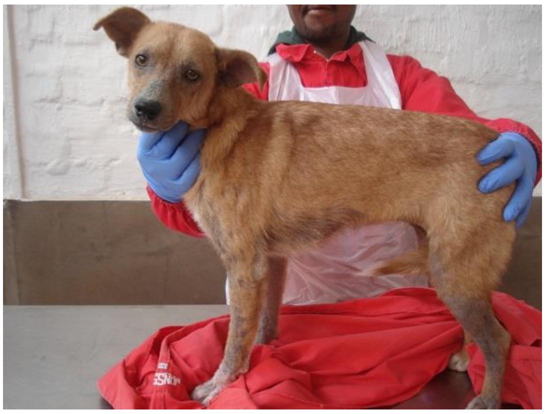

Day	Pre-treatment
Mite count	1832

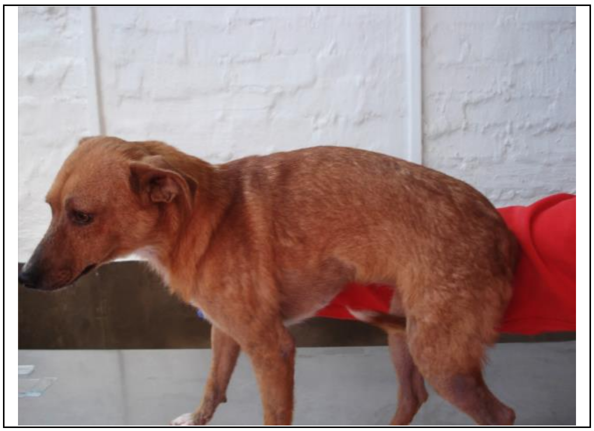

Day	28
Mite count	0

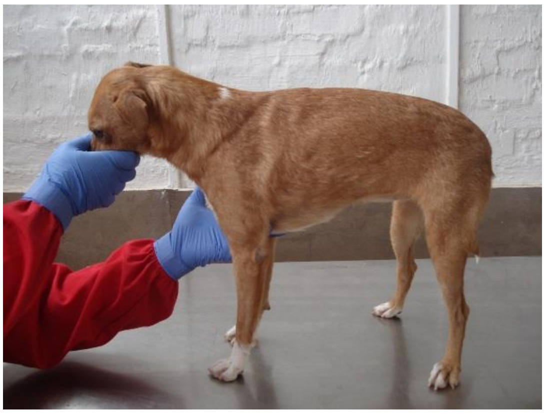

Day	56
Mite count	0

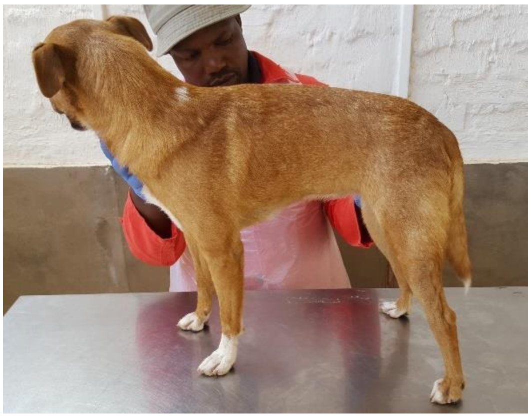

Day	84
Mite count	0

PHOTOGRAPHIC DOCUMENTATION

Animal ID	57B 205*
Treatment	NexGard®

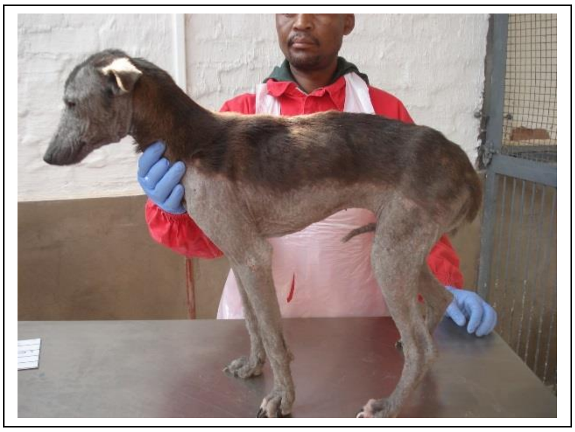

Day	Pre-treatment
Mite count	676

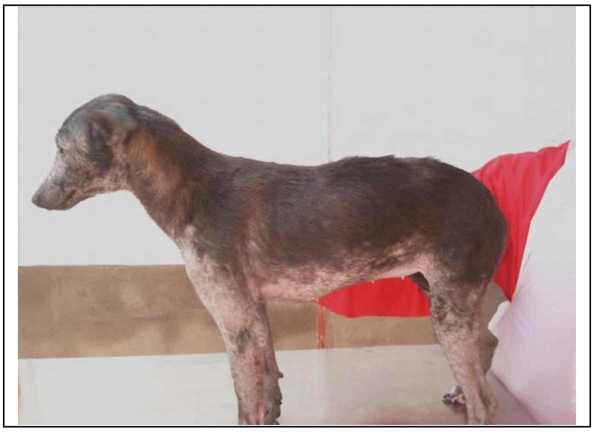

Day	28
Mite count	25

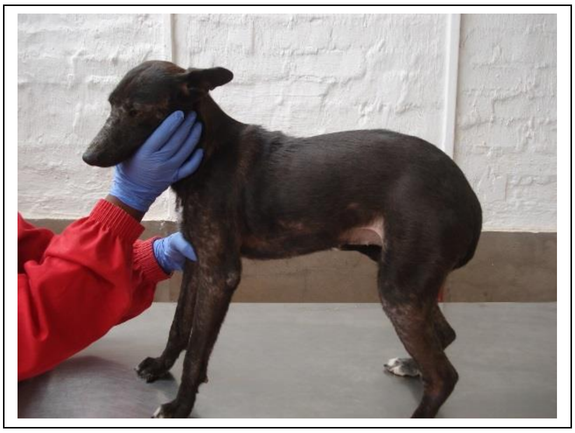

Day	56
Mite count	0

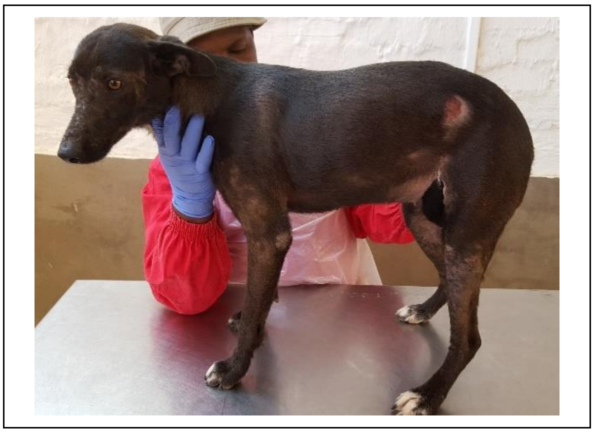

Day	84
Mite count	0

* This particular case shows slow clinical recovery, illustrating that some dogs may need longer clinical follow-up and treatment duration (e.g. 3 months or more).
